# Oxidation and allocation of nectar amino acids during butterfly flight

**DOI:** 10.1242/jeb.251674

**Published:** 2026-02-09

**Authors:** Natasha Tigreros, Goggy Davidowitz, Chloe Burkholder, Chloé Chabaud

**Affiliations:** ^1^ Department of Entomology, University of Arizona, Tucson, AZ 85719, USA; ^2^ Department of Ecology and Evolutionary Biology, University of Arizona, Tucson, AZ 85719, USA

**Keywords:** Nectar chemistry, Trade-offs, Pollinators, Nutrition, Stable isotopes, Proline

## Abstract

Flying animals face extreme energetic demands, relying mainly on carbohydrates and lipids, with occasional contributions from proteins and amino acids. In nectar-feeding species such as butterflies and hummingbirds, sugars are the primary fuel, yet the extent to which nectar-derived amino acids support flight versus other functions remains unclear. Using ^13^C-labelled nectar, we tracked the metabolic fate of sugars and amino acids during flight in *Pieris rapae* butterflies. We found that proline and glycine, two abundant nectar amino acids, were oxidized alongside sugars. We also compared females subjected to low- versus high-intensity flight. High flight intensity females incorporated less glycine into tissues, implying greater diversion toward energy use during flight. In contrast, they deposited more threonine – an essential amino acid – into their abdomens, prioritizing reproduction and storage. These findings reveal the role of nectar-derived nutrients in supporting locomotion and reproduction, while showing how nectar use can modulate trade-offs between flight and fecundity.

## Teaser

**Summary:** Nectar-derived amino acids can directly fuel butterfly flight and intense flight shifts how these nutrients are incorporated into the body, revealing a mechanism linking nectar composition, flight energetics and reproductive investment in pollinating insects.

## INTRODUCTION

Powered flight is a key adaptation across animals, allowing insects, birds and bats to forage, escape predators, locate mates and disperse over long distances (Anderson and Ruxton, 2020; Dudley, 2002; Ruaux et al., 2020). Yet, flight is also the most energetically costly mode of locomotion, far exceeding the metabolic demands of running or swimming (Bale et al., 2014; Schmidt-Nielsen, 1972). In insects, for example, flight can demand up to 100 times the resting metabolic rate (Dudley, 2002). Thus, meeting these energetic demands requires efficient fuel use, with flying animals relying primarily on carbohydrates and lipids, and, in some cases, proteins and amino acids. The relative contribution of each fuel depends on the species, flight intensity, ecological context and resource availability (Weber, 2011).

For nectar-feeding animals such as butterflies, bees and hummingbirds, nectar sugars are the dominant energy source during flight (Abdul Razak et al., 2011; Suarez et al., 2011). Yet, nectar is a complex resource that also contains small but physiologically relevant amounts of amino acids (Baker and Baker, 1986; Nicolson and Thornburg, 2007). While studies, especially in insects, indicate that some amino acids are oxidized from internal stores during flight (Teulier et al., 2016; Stec et al., 2021; Weeda et al., 1979), it is still unclear whether amino acids obtained directly from nectar are similarly used in flight.

Beyond serving as a fuel for flight, nectar can also contribute to reproduction and long-term somatic functions. In many butterflies and moths, for example, nectar sugars as well as amino acids are incorporated into proteins and directly support oogenesis, supplementing or in some cases even replacing nutrient reserves acquired during larval development (Jervis and Boggs, 2005; Levin et al., 2017; Mevi-Schütz and Erhardt, 2005). Similarly, in bird pollinators, especially those that rely heavily on nectar, amino acids in nectar have been suggested to help supplement their nitrogen requirements (Nicolson, 2007; Roguz et al., 2019). This dual role of nectar-derived resources may accentuate tradeoffs between flight and reproduction, as amino acids oxidized to sustain flight are no longer available for egg production. Thus, understanding whether nectar amino acids are metabolized during flight, or instead retained for tissue incorporation, is critical to link flight energetics with fitness and help clarify the physiological mechanisms underlying frequently observed trade-offs between flight and reproduction (Schmidt-Wellenburg et al., 2008; Tigreros and Davidowitz, 2019).

Here, we investigated the metabolic fate of nectar-derived nutrients in *Pieris rapae*, a common butterfly that relies on floral nectar throughout its adult life. Using ^13^C-labelled sugars and amino acids (proline, glycine and threonine), we examined nutrient oxidation during flight as well as nutrient deposition in thoracic and abdominal tissues under varying flight conditions. By distinguishing which nectar components serve as immediate fuels and which are retained for other functions, we provide new insight into the role of nectar composition in supporting the energetics of insect flight.

## MATERIALS AND METHODS

### Study organism and rearing

The cabbage white butterfly, *Pieris rapae* (Linnaeus 1758), is a widespread species whose adults feed on floral nectar, serving as pollinators for a variety of plants while obtaining sugars and amino acids as nutritional rewards (Alm et al., 1990; Corbera et al., 2018; Hongfang et al., 2010; Rader et al., 2009). Like other pollinators, female *P. rapae* can detect and preferentially consume nectars enriched in amino acids (Alm et al., 1990). This species faces high energetic demands due to its investment in both flight and reproduction: females fly an average of 0.7 km per day (Scott, 1987) and lay an average of 278±172 eggs over a short adult lifespan of about 12.2±4.0 days (means±s.d.) (Kimura and Tsubaki, 1986).

For this study, *P. rapae* were obtained from a laboratory colony established from a wild population in UT, USA. Larvae were reared in a greenhouse on *Brassica oleracea*. Pupae and adults were maintained in a walk-in environmental chamber at 22°C and 50% relative humidity (RH) on a 16 h:8 h light:dark cycle.

### Nectar treatments

In this study, we used artificial nectars that replicated the sugar and amino acid composition of *Lantana camara* (Verbenaceae), a high-quality nectar source frequently visited by diverse butterfly species. The composition of *L. camara* nectar has been well characterized and is widely used in studies of nectar preference in *P. rapae* (Alm et al., 1990) and other Lepidoptera (Erhardt and Rusterholz, 1998; Jervis and Boggs, 2005; Mevi-Schütz and Erhardt, 2003). Natural *L. camara* nectar typically contains ∼0.547 mol l^−1^ sucrose and ∼10 mmol l^−1^ amino acids, yielding approximately a 1:100 amino acid to sugar ratio. Of the amino acid pool, roughly 6% comprises three essential amino acids, with threonine (0.672 mmol l^−1^) being the most abundant, while the remaining 94% consists of eight non-essential amino acids, dominated by proline (2.23 mmol l^−1^) and glycine (2.37 mmol l^−1^). For the full nectar composition, see Table S1. To track use of specific nectar nutrients by female *P. rapae*, we created five *Lantana*-mimicking nectar treatments. Four contained a single ^13^C-labelled compound – ^13^C_1_-glycine, U^13^C-proline, ^13^C_1_-threonine or ^13^C-sucrose (enriched with cane sugar) – while the fifth served as a control, containing beet sugar and only unlabelled amino acids. The labelled amino acids were selected because they are abundant in *L. camara* nectar and in other butterfly-pollinated flowers (Mevi-Schütz and Erhardt, 2003; Roguz et al., 2019). Glycine and threonine were labelled at the carboxyl carbon (^13^C_1_), which is lost immediately as CO_2_ during amino acid catabolism and thus produces strong signals in breath analysis (see experiment 1) but can result in lower tissue enrichment (see experiment 2); proline, in contrast, was uniformly labelled (U-^13^C), across all five carbons, which can lead to large signals in both breath and tissues. Finally, sucrose was enriched with cane sugar (δ^13^C≈−10), differing from control with beet sugar (δ^13^C≈−26.5) due to natural C_4_–C_3_ differences (for details on nectar composition and ^13^C enrichment, refer to Table S1). All isotope tracers were obtained from Cambridge Isotope Laboratories, Inc. (Tewksbury, MA, USA). All δ^13^C values are reported relative to the international standard Vienna Pee Dee Belemnite (VPDB), meaning that δ^13^C indicates carbon isotope ratios expressed in per mille (‰) relative to this reference.

For all experiments, we provided butterflies with a fixed nectar volume rather than allowing *ad libitum* feeding, in order to standardize nutrient intake across individuals and treatments. Based on pilot trials, a 10 µl dose was selected as the largest volume that all females consistently consumed in a single feeding event under our laboratory conditions, thereby minimizing variation in intake unrelated to experimental factors.

### Experiment 1: oxidation of nectar nutrients during flight

To assess oxidation of nectar nutrients during flight, females were fed 48 h post-emergence with 10 µl of either a control nectar or one of four ^13^C-labelled nectars: ^13^C_1_-glycine, U^13^C-proline, ^13^C_1_-threonine or ^13^C-sucrose. Four hours after feeding, each female was placed in a respirometry chamber consisting of a 500 ml syringe fitted with an injection port and adjusted to provide a 200 ml chamber volume. This volume was sufficient to allow flight while enabling CO_2_ accumulation to ≥300 ppm, the minimum concentration recommended for reliable δ^13^C measurements (Picarro Inc. G2201-i user manual, see below). The syringe air was replaced with dried, CO_2_-free air, sealed, and females were flown for 4 min under natural light, with gentle shaking to prevent resting. A 20 ml breath sample was then analysed for δ^13^CO_2_ using a cavity ring-down spectrometer (G2201-i, Picarro Inc., Sunnyvale, CA, USA) coupled to a SSIM2 Small Sample Isotope Module (Picarro Inc.).

### Experiment 2: the effect of flight on nectar nutrient deposition

To determine where nectar-derived amino acids are deposited, we used a different group of adult females (48 h post-emergence) which were randomly assigned to one of two flight treatments – low or high flight intensity – and fed either a control nectar or one of four ^13^C-labelled nectars. In the low flight intensity treatment, females were kept in a 30×30×30 cm mesh cage lined with paper towel inside a climate-controlled walk-in chamber (16 h:8 h light:dark, 22°C, 50% RH). These females spent most of the day perched and performed only short, voluntary flights while repositioning. In the high flight intensity treatment, females were induced to fly for 5 min day^−1^ by gently touching them with a fine paintbrush (Niitepõld and Boggs, 2015). During the induced flight period, females flew almost continuously and showed clear signs of fatigue near the end of the session, indicating short-term exhaustion. Thus, high flight intensity butterflies experienced a consistent period of elevated energetic demand, whereas low flight intensity butterflies engaged in minimal flight and were never pushed to exhaustion. All females were weighed 24 h after emergence (to the nearest 0.001 mg, Sartorius Quintix65-1S-) and then fed once daily with 10 µl of their assigned nectar treatment. After three consecutive days of the flight and nectar treatments, individuals were euthanized by freezing at −20°C. Thoraces and abdomens were dissected, dried at 50°C for 48 h, and weighed separately using a high-precision microbalance (Sartorius Quintix65-1S; 0.001 mg resolution). These post-treatment dry masses provided a gravimetric estimate of thoracic and abdominal resource status in low versus high flight intensity females. Dried tissues were subsequently homogenized, and δ^13^C values were measured using cavity ring-down spectroscopy (CRDS; Picarro Inc. stable isotope analyser, G2201-i) coupled with an A0201 combustion module and A0301 gas interface.

### Statistical analysis

Experiment 1: to determine whether butterflies oxidized ^13^C-labelled nutrients during flight, we analysed δ^13^C_breath_ values using a linear model with nectar treatment (control, sugars, glycine, proline, threonine) as the fixed effect. Because our *a priori* interest was whether each labelled nectar differed from the unlabelled control, we used Dunnett-adjusted treatment–control contrasts. We report contrast estimates, standard errors and adjusted *P*-values.

Experiment 2: Before examining effects of the flight treatment, we assessed incorporation of nectar-derived nutrients into tissues, comparing δ^13^C_tissue_ values within each flight treatment and tissue type separately. As in experiment 1, inference focused on pre-planned comparisons of each ^13^C-labelled nectar with the unlabelled control, using Dunnett-adjusted contrasts. For each comparison, we report the contrast estimate, standard error and adjusted *P*-value.

Baseline δ^13^C values differ naturally among tissues because of differences in biochemical composition, and flight intensity can further shift these values by altering the relative oxidation of endogenous fuels with distinct isotopic signatures (e.g. lipids, carbohydrates, protein) (Mintenbeck et al., 2008). Consequently, to compare nectar use across tissues and flight treatments, we calculated atom percentage excess (APE), defined as APE=AP(label)−AP(control). Atom percentage (AP) values were derived from δ^13^C values of labelled and control tissues (expressed relative to VPDB) (McCue et al., 2011; Slater et al., 2001).

Note that absolute δ^13^C and APE values were not proportional across nutrients because each tracer differs in the number of labelled carbons and position, and in concentration within nectar (Table S1). Thus, statistical analysis and interpretation of results is based on quantitative inferences within each nutrient (labelled versus control; thorax versus abdomen; high versus low flight intensity), and cross-nutrient comparisons (e.g. glycine versus sucrose) can only be interpreted qualitatively in terms of whether a nutrient is oxidized or incorporated, or whether its incorporation changes with flight.

The effects of flight treatment on gravimetric estimates of thorax and abdomen mass were tested using an ANCOVA that included flight treatment as a fixed factor and initial female body size as a covariate. Allocation of nectar-derived nutrients to thoracic and abdominal tissues was analysed using two-way ANOVA with flight treatment, nectar nutrient treatment (^13^C-labelled versus control) and their interaction as fixed factors. When significant interactions were detected, we estimated predicted values and performed pairwise comparisons of simple effects using the *emmeans* package in R, applying a Tukey adjustment for multiple testing. All statistical analyses were conducted in R 3.6.0 (http://www.R-project.org/) and assumptions of normality and homogeneity of variance were verified prior to analysis.

## RESULTS AND DISCUSSION

Nectar is a critical resource for flying pollinators, providing the energy needed to support their high metabolic demands during flight (O'Brien, 1999; Suarez, 2005; Welch et al., 2006). While it is well established that nectar sugars serve as the primary fuel for flight, far less is known about the roles of non-sugar components, such as amino acids. Here, we investigated the use of nectar-derived nutrients, including essential and non-essential amino acids, as metabolic fuels during flight and how flight activity may influence their allocation to different tissues in female *P. rapae*.

### Oxidation of nectar nutrients during flight

**Fig. 1.**
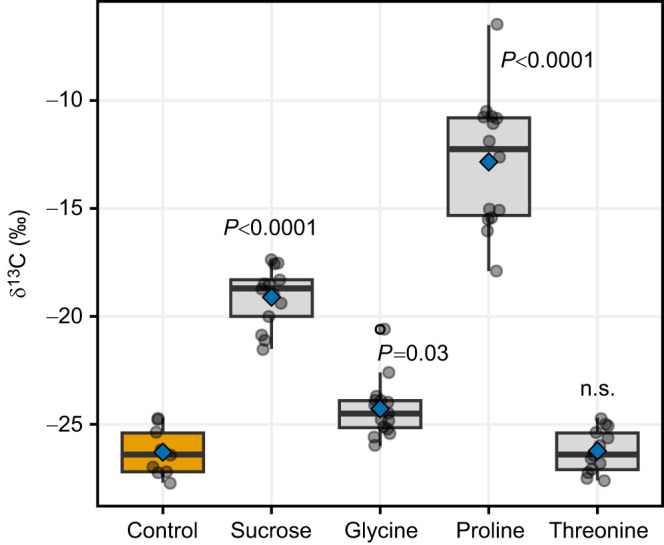
**δ^13^C values from exhaled breath during flight of *Pieris rapae* females.** A significant increase in δ^13^C in comparison with the control group (*n*=9; orange) indicates oxidation of a nectar-derived nutrient during flight. Labelled nutrients were sucrose (*n*=13), the two non-essential amino acids glycine (*n*=15) and proline (*n*=14), and an essential amino acid, threonine (*n*=13). Boxplots show the median, upper and lower quartiles and 1.5× the interquartile range; individual data points are shown as circles; mean values are indicated by blue diamonds. *P*-values correspond to Dunnett's tests comparing each labelled nutrient with the control (n.s., not significant). Note that because nectar concentration and ^13^C enrichment differ strongly among nutrients, δ^13^C magnitudes are not directly comparable across nutrients.

Females that fed on nectar containing ^13^C-labelled sucrose, proline or glycine showed significant ^13^CO_2_ enrichment (more positive δ^13^C values) in breath compared with controls fed unlabelled nectar (Fig. 1), indicating active oxidation of both sucrose and non-essential amino acids. Although previous studies have suggested that nectar amino acids help meet the high metabolic demands of flying nectarivores (Carter et al., 2006; Levin et al., 2017; Stec et al., 2021), our results provide the first direct evidence that nectar amino acids are actually oxidized during flight. At the same time, no significant oxidation of threonine by flying females was observed (Fig. 1), suggesting that essential amino acids may be less likely to contribute to locomotion, at least under our experimental conditions. These results add to the growing body of evidence highlighting the importance of nectar amino acids for flying animals, in particular for insect pollinators. Proline and glycine are common constituents of floral nectar (Baker and Baker, 1973; Rusterholz and Erhardt, 1998) and can act as a phagostimulant in pollinator insect species (Lim et al., 2019; Ruedenauer et al., 2019). Several studies have shown that proline, in particular, is selectively oxidized in insect flight muscle, especially during the initial 30 s of flight (Stec et al., 2021; Teulier et al., 2016; Weeda et al., 1979). Though glycine's role in flight is less defined, its consumption has been linked to neuromodulation and memory in insects (Parkinson et al., 2025), suggesting a dual function: fuelling flight and supporting cognitive processes essential for effective foraging.

### Effect of flight on nectar nutrient deposition

**Fig. 2.**
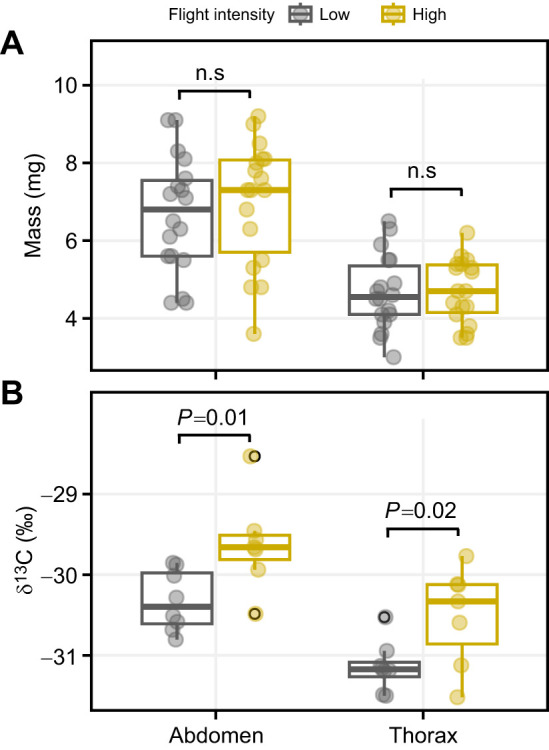
**Effects of flight intensity on dry mass and δ**^13^**C values of the same tissues in *P. rapae* females.** (A) Post-treatment dry mass of thorax and abdomen tissues from the same individuals subjected to low (*n*=18) and high flight intensity (*n*=18) treatments. (B) δ^13^C values of thorax and abdomen tissues from control females fed unlabelled nectar; these values reflect inherent biochemical contrasts among tissues (e.g. lipid-rich tissues tend to be relatively ^13^C depleted) as well as variation in fuel use associated with flight activity (high flight intensity, *n*=7; low flight intensity, *n*=8). Flight-treatment effects were tested using linear models, and *P*-values shown correspond to the flight treatment term for each tissue. Boxplots show the median, upper and lower quartiles and 1.5× the interquartile range; individual data points are shown as circles.

Gravimetric measurements of thorax and abdomen mass, controlling for adult body size, did not differ between flight treatments (ANCOVA: thorax, *F*_1,33_=0.13, *P*=0.71; abdomen, *F*_1,33_=0.07, *P*=0.13; Fig. 2A), indicating that 3 days of sustained flight were not sufficient to produce detectable changes in bulk tissue mass. In contrast, isotopic analysis of control females (fed unlabelled nectar) revealed baseline differences in δ^13^C among tissues and between females experiencing different flight intensities (Fig. 2B). Abdominal tissue showed higher (less negative) δ^13^C values than thoracic tissue (ANOVA: *F*_1, 26_=8.1, *P*=0.01), reflecting inherent biochemical differences between the abdomen and the thorax. Importantly, females exposed to high flight intensity exhibited higher δ^13^C values in both tissue types than those of low flight intensity females (thorax, *F*_1,13_=6.5, *P*=0.02; abdomen, *F*_1,14_=7.9, *P*=0.01; Fig. 2B), indicating increased reliance on endogenous fuels that differ in their isotopic signatures. Because lipids are naturally ^13^C depleted relative to proteins and structural carbohydrates, changes in δ^13^C are consistent with intense locomotion causing greater depletion of lipid stores, leaving tissues proportionally enriched in components with higher δ^13^C signatures (DeNiro and Epstein, 1977; McCue et al., 2015; Mintenbeck et al., 2008). This is in line with previous studies showing that butterflies rely on carbohydrates as well as lipids to meet the high energy costs of flight, and that sustained flight can rapidly deplete internal energy, often leading to flight fecundity tradeoffs (Tigreros and Davidowitz, 2019).

**Fig. 3.**
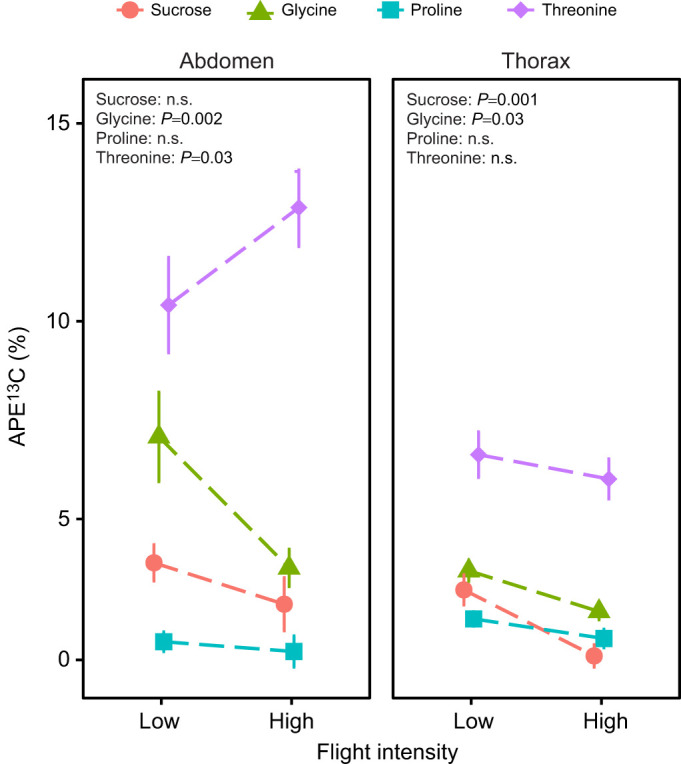
^13^**C enrichment in abdomen (left) and thorax (right) tissues of *P. rapae* females that consumed** ^13^**C-labelled nectar under low and high flight intensity treatments.** ^13^C enrichment is shown as atom percentage excess (APE). Points and lines represent means±s.e.m. Low versus high flight intensity comparisons for each nutrient (sucrose, glycine, proline, threonine) are indicated at the top of each panel. Because nectar concentration and ^13^C enrichment differ strongly among nutrients, APE magnitudes are not directly comparable across nutrients. Effects of flight treatment and nutrient type were evaluated using two-way ANOVA, followed by simple-effects tests for low versus high contrasts within each nutrient, and *P*-values reported reflect these simple-effects comparisons.

Previous studies have shown that nectar in general and its amino acids in particular can be rapidly incorporated, within minutes, into Lepidoptera flight muscle and developing oocytes (DeFino and Davidowitz, 2024; Levin et al., 2017). Given that the thorax serves as the primary site of fuel combustion during flight (Dudley, 2002; Treidel et al., 2024), it can be expected that incoming amino acids and sugars are preferentially oxidized rather than retained. Here, we demonstrate that flight activity significantly alters the allocation of nectar nutrients across female tissues (*F*_3,102_=5.33, *P*=0.002). In the thorax, the presence of nectar-derived nutrients showed an overall decline with increasing flight intensity (*F_1,51_*=4.99, *P*=0.03; Fig. 3; Table S2A), consistent with increased metabolic oxidation or mobilization under high flight demand. However, simple-effects comparisons revealed that this decrease was statistically significant only for sucrose and glycine (Table S2B). Notably, although most labelled amino acids were detected in both thorax and abdomen tissues (Fig. S1), proline was not detected in the thorax under high flight intensity conditions, and it only approached significance in low flight intensity females (*P*=0.09; Fig. S1). This supports the hypothesis that proline is rapidly oxidized during flight, leaving little available for tissue incorporation.

Patterns of nutrient incorporation and use were only distinct in the abdomen, reflecting its key roles in nutrient storage and reproduction. We found that changes in the incorporation of nectar nutrients with flight varied depending on nutrient type (*F*_3,51_=4.9, *P*=0.01): glycine decreased (*P*=0.002), while threonine increased (*P*=0.03) with higher flight activity (Fig. 3; Table S2). Interestingly, proline and sucrose incorporation remained unchanged (Fig. 3; Table S2). As the primary site of nutrient storage and reproduction, the abdomen likely channels nectar-derived nutrients toward oogenesis and long-term metabolic demands (Boggs, 2009; O'Brien et al., 2002). The decline in abdominal glycine, an amino acid significantly oxidized during flight (Fig. 1), suggests that this non-essential amino acid may be diverted to meet immediate energy demands, possibly contributing to a trade-off with female fecundity. Conversely, the increase in threonine, an essential amino acid, may reflect its selective prioritization for egg provisioning. These findings support the idea that adult females use nectar to buffer against nutrient limitations, whether from poor larval diets (Mevi-Schütz and Erhardt, 2005; O'Brien et al., 2000) or from increased metabolic demands from flight.

Collectively, our findings demonstrate that a flying nectarivorous insect utilizes both sugars and specific amino acids – particularly proline and glycine – as metabolic fuels during flight. Moreover, intense flight alters the fate and allocation of nectar-derived nutrients in adult tissues, likely reflecting a trade-off between immediate energy needs and resource storage. Although direct evidence in nectarivorous vertebrates is scarce, the high concentrations of amino acids (including proline and glycine) in nectar of some bird-pollinated plants suggest these compounds may serve similar functions in these species (Nicolson, 2007; Roguz et al., 2019). Thus, our results provide broader insights into how nectar composition can influence locomotion and fitness of nectar-feeding species, emphasizing the ecological significance of amino acids in adult diets, especially under conditions of elevated energy expenditure.

## Supplementary Material

10.1242/jexbio.251674_sup1 Supplementary information
