# Supplementary material for: Oxidation and allocation of nectar amino acids during butterfly flight
Source: J Exp Biol. 2026 Feb 9;229(3):jeb251674. doi: 10.1242/jeb.251674 (PMC12951608; doi:10.1242/jeb.251674)
Supplement: Supplementary information [file jexbio-229-251674-s1.pdf]

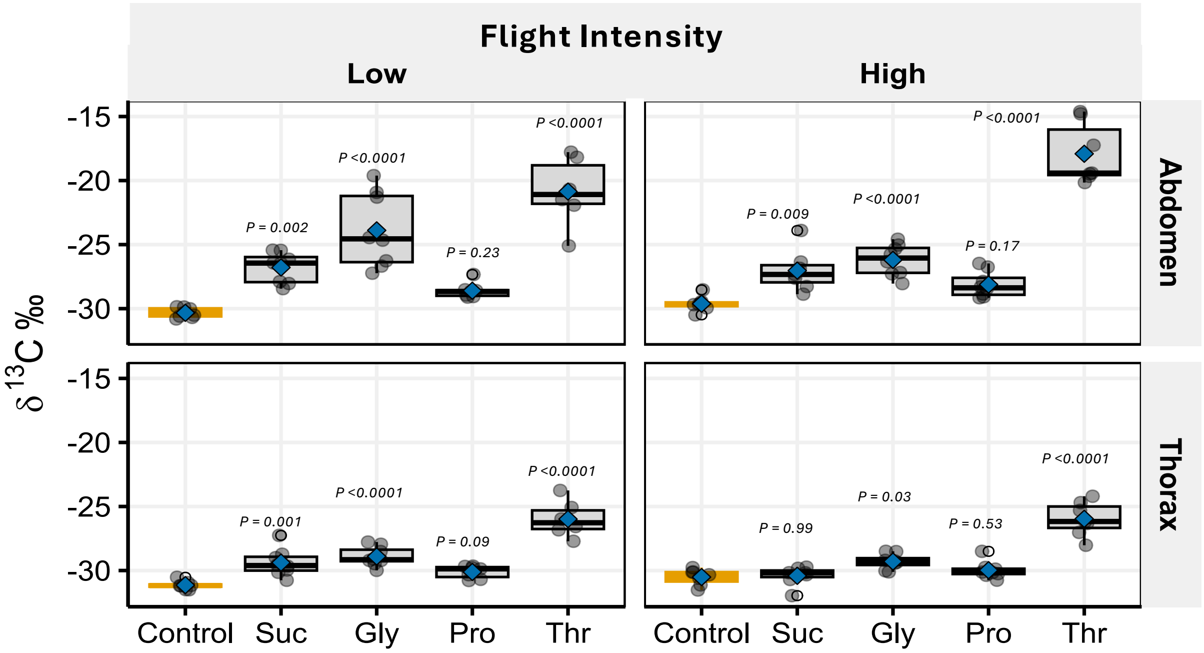

Fig. S1. $\delta^{13}C$ values of thorax and abdomen tissues of female *Pieris rapae* fed ^{13}C-labelled nectars under Low- and High-flight treatments. Boxplots (with arithmetic means shown as blue diamonds) depict $\delta^{13}C$ values for females fed either unlabeled control nectar or nectars labelled with sucrose (Suc), glycine (Gly), proline (Pro), or threonine (Thr). The figure is divided into four panels by tissue type (thorax, abdomen) and flight intensity (Low, High). *P*-values shown in each panel correspond to Dunnett's tests comparing each labelled nutrient (grey boxplots) with its respective control (yellow boxplot).

Journal of Experimental Biology • Supplementary information

Table S1. Composition, carbon dose, and effective ^{13}C enrichment of sucrose, glycine, proline, and threonine traced in the nectar treatments. Differences in nectar concentration and labeling architecture produce large differences in carbon dose (concentration × molecular carbon content) and effective ^{13}C atoms per molecule. Sucrose, present at 0.547 M, provides an extremely high carbon dose but only minimal natural C_4-derived ^{13}C enrichment (~0.002 labeled carbons per molecule). In contrast, amino acids occur at millimolar concentrations but carry much stronger labels, with glycine and threonine each labeled at the carboxyl carbon (1 labeled C) and proline uniformly labeled (5 labeled C). Consequently, $\delta^{13}C$ and APE magnitudes differ inherently across nutrients and should be interpreted quantitatively only within each nutrient treatment.

Nutrient	Nectar Concentration	Label Type	Total C Atoms	Effective ^{13}C atoms per molecule	C-atoms per liter in nectar	Relative C dose	Relative ^{13}C Enrichment
**Sucrose (cane)**	0.547 M	Natural C_4 enrichment	12	~0.002	0.547 × 12 = **6.56 mol C/L**	Exptremely High	Low
**Glycine**	2.371 m	C1-labeled	2	1 labeled C	2.371×2 = **4.74 mmol C/L**	Low	High
**Proline**	2.23 m	Uniform (U-^{13}C)	5	5 labeled C	2.23×5 = **11.15 mmol C/L**	High	Very High
**Threonine**	0.672 m	C1-labeled	4	1 labeled C	0.672×4 = **2.69 mmol C/L**	Very Low	Hig

Note: Additional nectar constituents not used as tracers included glucose (0.282 M), fructose (0.316 M), serine (1.37 mM), glutamine (0.931 mM), arginine (0.201 mM), and valine (0.137 mM).

Table S2. (A) Statistical output from general linear models (Anova type III) on the effects of flight intensity (Low vs. High), nectar nutrient, and their interaction, on atom percent excess (APE) values in thorax and abdomen tissues of *Pieris rapae* females. Because nectar concentration and ^{13}C enrichment differ among nutrients, the main effect of Nutrient is not biologically meaningful and should not be interpreted. In contrast, the Flight × Nutrient interaction is biologically relevant, as it indicates whether high-intensity flight alters the incorporation of nectar-derived nutrients differentially across nutrient types (e.g., glycine vs. proline). Degrees of freedom are shown in brackets [d.f.]. (B) Simple-effects pairwise comparisons evaluating the effect of flight intensity within each nutrient treatment for thorax and abdomen tissues. Estimates, standard errors, and adjusted *P*-values are reported.

(A) Anova Summary Table

	Abdomen		Thorax	
	F	*P-value*	*F*	*P-value*
Flight [1, 51]	10.60	< 0.01	4.99	0.03
Nutrient [3, 51]	22.16	< 0.0001	24.65	< 0.0001
Flight x Nutrient [3, 51]	4.90	< 0.01	1.21	0.31

(B) Simple effects High vs Low flight intensity

	Abdomen		Thorax	
	estimate	*P-value*	*estimate*	*P-value*
Sucrose	1.05±1.05	0.32	1.82±0.52	0.001
Glycine	3.33±1.02	0.002	1.13±0.5	0.03
Proline	0.25±1.05	0.81	0.54±0.52	0.30
Threonine	-2.47±1.13	0.03	0.67±0.56	0.23
